# Endothelial β-Catenin Deficiency Causes Blood-Brain Barrier Breakdown *via* Enhancing the Paracellular and Transcellular Permeability

**DOI:** 10.3389/fnmol.2022.895429

**Published:** 2022-05-09

**Authors:** Basharat Hussain, Cheng Fang, Xiaowen Huang, Ziying Feng, Yuxuan Yao, Yu Wang, Junlei Chang

**Affiliations:** ^1^Shenzhen Key Laboratory of Biomimetic Materials and Cellular Immunomodulation, Institute of Biomedicine and Biotechnology, Shenzhen Institute of Advanced Technology, Chinese Academy of Sciences, Shenzhen, China; ^2^University of Chinese Academy of Sciences, Beijing, China; ^3^State Key Laboratory of Pharmaceutical Biotechnology, Department of Pharmacology and Pharmacy, Li Ka Shing Faculty of Medicine, The University of Hong Kong, Pok Fu Lam, Hong Kong SAR, China

**Keywords:** blood-brain barrier, Wnt signaling, endothelial cells, tight junctions, transcytosis

## Abstract

Disruption of the blood-brain barrier (BBB) causes or contributes to neuronal dysfunction and several central nervous system (CNS) disorders. Wnt/β-catenin signaling is essential for maintaining the integrity of the adult BBB in physiological and pathological conditions, including stroke. However, how the impairment of the endothelial Wnt/β-catenin signaling results in BBB breakdown remains unclear. Furthermore, the individual contributions of different BBB permeability-inducing mechanisms, including intercellular junction damage, endothelial transcytosis, and fenestration, remains unexplored. Here, we induced β-catenin endothelial-specific conditional knockout (ECKO) in adult mice and determined its impact on BBB permeability and the underlying mechanism. β-catenin ECKO reduced the levels of active β-catenin and the mRNA levels of Wnt target genes in mice, indicating downregulation of endothelial Wnt/β-catenin signaling. β-catenin ECKO mice displayed severe and widespread leakage of plasma IgG and albumin into the cerebral cortex, which was absent in wild-type controls. Mechanistically, both the paracellular and transcellular transport routes were disrupted in β-catenin ECKO mice. First, β-catenin ECKO reduced the tight junction protein levels and disrupted the intercellular junction ultrastructure in the brain endothelium. Second, β-catenin ECKO substantially increased the number of endothelial vesicles and caveolae-mediated transcytosis through downregulating Mfsd2a and upregulating caveolin-1 expression. Interestingly, fenestration and upregulated expression of the fenestration marker Plvap were not observed in β-catenin ECKO mice. Overall, our study reveals that endothelial Wnt/β-catenin signaling maintains adult BBB integrity *via* regulating the paracellular as well as transcellular permeability. These findings may have broad applications in understanding and treatment of CNS disorders involving BBB disruption.

## Introduction

The central nervous system (CNS) is separated from the blood circulation by a special interface called the blood-brain barrier (BBB). The BBB maintains the CNS microenvironment homeostasis by restraining the entry of neurotoxic substances and pathogens. The BBB also allows the selective influx of nutrients and hormones, and the efflux of metabolic wastes, to facilitate proper neuronal functions (Profaci et al., 2020). BBB disruption can cause or contribute to neuronal dysfunction and many CNS disorders, including stroke, brain tumors, epilepsy, multiple sclerosis, and Alzheimer’s disease (Sweeney et al., 2018; Profaci et al., 2020; Hussain et al., 2021). Furthermore, BBB breakdown has been implicated in the neurological complications of systemic inflammation, such as bacterial and severe acute respiratory syndrome coronavirus 2 (SARS-CoV-2) infection (Huang et al., 2021).

Blood-brain barrier integrity is collectively determined by a multicellular microstructure consisting of endothelial cells (ECs), pericytes, astrocytes, neurons, and the extracellular matrix connecting them; all these components collectively form the neurovascular unit (NVU) (Iadecola, 2017). Microglia and oligodendrocyte progenitor cells also have been indicated to regulate BBB integrity (Seo et al., 2014; Ronaldson and Davis, 2020). Among the different components of the NVU, ECs serve at the frontline, facing the bloodstream and thus, play a dominant role in determining BBB permeability. Brain ECs control paracellular and transcellular passage pathways through two unique features: increased expression of tight junction (TJ) proteins to preclude paracellular passage of blood-borne molecules and cells, and restricted transcytosis and fenestration to restrain non-specific transcellular transport of blood contents (Langen et al., 2019). Instead, the nutrients and hormones essential for brain function are transported across the BBB by specific transporters and receptors expressed in brain ECs. Decreased TJ protein levels or increased endothelial transcytosis are often observed in various CNS diseases, and both can lead to leakage of neurotoxic blood substances into the brain parenchyma (Liebner et al., 2018; Profaci et al., 2020).

The endothelial Wnt/β-catenin signaling pathway plays an essential role in BBB formation and maintenance in health, as well as in diseases such as ischemic stroke, glioblastoma, medulloblastoma, and multiple sclerosis (Liebner et al., 2008; Zhou et al., 2014; Phoenix et al., 2016; Chang et al., 2017; Lengfeld et al., 2017; Niu et al., 2019; Ta et al., 2021). In mice, deletion of genes encoding the molecules participating in Wnt/β-catenin signaling pathway results in severe BBB breakdown during embryonic development or adulthood (Stenman et al., 2008; Daneman et al., 2009; Kuhnert et al., 2010; Wang et al., 2012; Zhou et al., 2014; Tran et al., 2016). However, the mechanisms underlying the impairment of endothelial Wnt/β-catenin signaling leading to BBB breakdown remain unclear. Previous studies have demonstrated that decreased Wnt/β-catenin signaling causes increased paracellular permeability of the BBB by downregulating the expression of TJ proteins and disruption of intercellular junction ultrastructure (Zhou et al., 2014; Tran et al., 2016). Nevertheless, the role of endothelial transcytosis and fenestration in regulating paracellular permeability of the BBB has not been explicitly determined.

Here, we first generated an adult mouse model with endothelial-specific deletion of β-catenin, and then comprehensively examined the cellular, molecular, and ultrastructural alterations of the BBB upon impairment of endothelial Wnt/β-catenin signaling. Our results demonstrated that suppressed endothelial β-catenin signaling increased paracellular and transcellular permeabilities by damaging the intercellular junctions, and by substantially increasing the caveolae-mediated transcytosis. However, fenestration was not observed in brain ECs. Our findings provide mechanistic insights into the regulation of BBB integrity by Wnt/β-catenin signaling and have broad applications in understanding and treatment of CNS disorders involving BBB disruption.

## Materials and Methods

### Antibodies, Ligands, and Chemicals

The antibodies, ligands, and chemicals used in this study are listed in Supplementary Table 1.

### Cell Culture

Mouse brain microvascular endothelial cells (bEnd.3) were purchased from the ATCC cell bank (American Type Culture Collection, CRL-2299) and grown in DMEM (Dulbecco’s Modified Eagle Medium) supplemented with 10% serum and antibiotics, in a humidified incubator supplied with 5% CO_2_ and set at 37°C. After no more than three passages, cells at 80–90% confluency were seeded in a 12-well plate. The cells were serum-starved for 12 h and treated with different concentrations of Wnt3a and LiCl for 24 h.

### Tamoxifen Preparation and Administration

Ethanol and sunflower seed oil were used to dissolve tamoxifen (Solarbio, Beijing, China) by sonication (2 mg/ml) for immediate use or stored at –20°C. Tamoxifen was administered orally to all mice (2 mg/10 g body weight, four times every other day).

### Animal Model

The animals were bred at the specific pathogen free animal care houses. The research protocols and the use of animals were approved by the ethical committee of Shenzhen Institute of Advanced Technology, Chinese Academy of Sciences, Shenzhen, China.

The *Ctnnb1* (β-catenin) flox mice and *Cdh5-CreER* mice were generated as previously described (Brault et al., 2001; Wang et al., 2010). *Ctnnb1*^loxp/loxp^*; Cdh5-CreER^–^* mice were crossed with *Ctnnb1*^loxp/loxp^*; Cdh5-CreER^+^* mice to generate endothelial cell (EC) β-catenin knockout mice *Ctnnb1*^loxp/loxp^*; Cdh5-CreER^+^* (β-cat ECKO) and *Ctnnb1*^loxp/loxp^*; Cdh5-CreER^–^* control mice (β-cat WT). Male 8–10-week-old mice were used and sacrificed at 2–3 days after the last dose of tamoxifen was administered.

### Real-Time Quantitative PCR

Total RNA extraction from whole mouse brains as well as bEnd.3 cells treated with Wnt3a and/or LiCl by using TRIzol^®^ reagent (Thermo Fisher, Waltham, MA, United States) following manufacturer’s protocol. Complementary DNA (cDNA) was generated by performing real-time quantitative PCR (RT-qPCR) using 200 ng RNA and iScript Reverse Transcription Supermix (Vazyme, Nanjing, China). To perform RT-qPCR cDNA (0.5 μg), specific primers and SYBR Green qPCR Master Mix were used. A StepOnePlus Real-Time PCR System (Roche, Switzerland) was used for RT-qPCR. Relative mRNA levels were quantified using *Ct* values and 2^–ΔΔCt^ method after normalizing their expression to that of β-actin. The sequence-specific PCR primers used in this study are listed in Supplementary Table 2.

### Western Blotting

Radio-immunoprecipitation assay buffer (Solarbio, Beijing, China) containing protease and phosphatase inhibitor was used to lyse the whole cell lysate and mouse brain tissue. The protein concentrations were quantified using the standard BCA (Solarbio, Beijing, China) (Bicinchoninic Acid; Bradford assay). Equal protein amount from each sample was separated by performing SDS-PAGE and using 8–10% resolving gels. The resolved proteins were then transferred onto polyvinylidene difluoride membranes (PVDF). Non-fat milk solution (5%) was used to block the membranes for 1 h at room temperature (RT). The membrane was then incubated with primary antibodies (listed in Supplementary Table 1) at 4°C overnight and washed five times with TBST, each time 5 min. Horseradish peroxidase (HRP)-conjugated respective secondary antibodies (Jackson ImmunoResearch, West Grove, PA, United States 1:10,000) were added to the membrane, which was then incubated for 1 h at RT and washed again with TBST as mentioned earlier. Chemiluminescent HRP substrate (Millipore; WBKLS0500) and GelView 6000M system (BioLight, Zhuhai, China) were used to detect the target protein bands. The ImageJ software was used to quantify the protein bands.

### Mouse Brain Tissues Preparation

Vascular endothelial cells of β-cat WT and β-cat ECKO mice model were used to determine immune reactivity in vascular endothelial cells for active (non-phosphorylated) β-catenin, Claudin-5, Occludin, ZO-1 (Zona occludens 1), Mfsd2a, Plvap, Caveolin-1, IgG, and Albumin using immunofluorescence staining (IF). 10% ketamine and xylazine were used as anesthetic agens for the mice, and ice-cold phosphate-buffered saline (PBS) was used for cardiac perfusion. Brain tissues were collected and fixed in 4% paraformaldehyde (PFA) for 1 h at RT. Brain tissues were first dehydrated in 15% sucrose at 4°C overnight, followed by 3–6 h incubation in 30% sucrose at 4°C. Brain tissues were then embedded in optimal cutting temperature (OCT) compound for 10–15 min at RT, and stored at –20 or –80°C. To stain PFA fixation-sensitive antibodies (Claudin-5, ZO-1), fresh brain tissues were embedded in OCT and placed in a –80°C freezer utill being used.

### Immunofluorescence Staining

Frozen brain sections of 10 μm thickness were incubated with blocking buffer [10% normal goat serum (Thermo Fisher, Waltham, MA, United States) + 0.2% Triton X-100] for 1 h at RT. The sections were then incubated with primary antibodies (listed in Supplementary Table 1) at 4°C overnight. The sections were washed four times with 1× PBS at RT, each wash lasting 15 min. Subsequently, the brain sections were incubated with fluorescently labeled secondary antibodies (Jackson ImmunoResearch, West Grove, PA, United States) for 2 h at RT. Brain sections were again washed four times with 1× PBS at RT, as described before. To stain the nuclei, the brain tissues were mounted with antifade medium containing DAPI (Solarbio, Beijing, China), and observed under a fluorescence microscope (Olympus, CKX53SF, Japan). ImageJ software was used to quantify the immunofluorescence signal area, which was normalized to the area of CD31 signal.

### Transmission Electron Microscopy

Transmission electron microscopy was used to observe the ultrastructural differences between the brain ECs of β-catenin WT and ECKO mice. For TEM analysis, brains were collected from 8 to 10 weeks mice (WT and ECKO) immediately after sacrifice without cardiac perfusion and fixed in 2.5% glutaraldehyde/0.1 M PBS (pH 7.2) at 4°C overnight. Graded ethanol was used for tissue dehydration, and epoxy resin was used to embed the dehydrated brain tissues. Uranyl acetate and lead citrate were then used to stain the ultrathin sections (80 nm) collected on copper grids. JEM-1400Plus TEM (JEOL, Tokyo, Japan) was used to analyze the ultrastructure of brain microvessels. To quantify vesicles and calculate the percentage of damaged adherens junctions and tight junctions per mouse, at least five microvessels were examined in the cerebral cortex of each mouse.

### Statistical Analysis

All results were obtained from at least three independent experiments. GraphPad Prism 6.01 was used to analyze the data. Data are presented as the mean ± SD. Variations between the two groups were analyzed by using two-tailed unpaired *t-*tests. Statistical significance was set at *P* values < 0.05.

## Results

### Endothelial Cell-Specific β-Catenin Deletion Suppresses Endothelial Wnt/β-Catenin Signaling in Adult Mice

**FIGURE 1:**
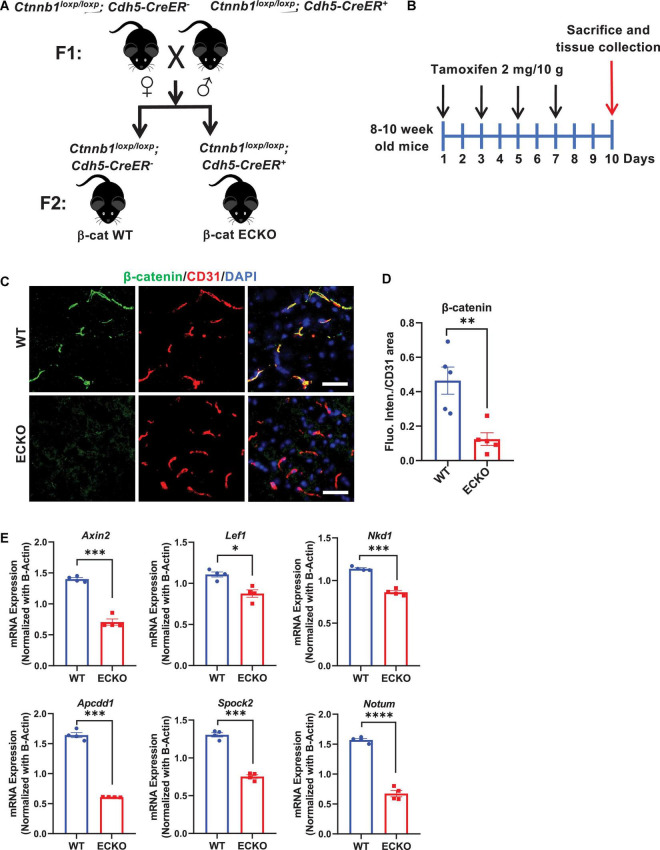
Endothelial cell-specific deletion of β-catenin suppresses the activity of endothelial Wnt/β-catenin signaling in adult mice. **(A)** Crossing of mice carrying *Ctnnb1*^loxp/loxp^*; Cdh5-CreER^–^* allele with mice harboring *Ctnnb1*^loxp/loxp^*; Cdh5-CreER^+^* allele to produce β-cat ECKO mice and β-cat WT control, respectively. **(B)** Schematic of the experiment design. **(C)** Immunofluorescence staining showing the expression pattern and levels of active β-catenin (non-phosphorylated form) in the cerebral cortex from β-cat WT mice and β-cat ECKO mice, respectively. Scale bars: 100 μm. **(D)** Quantification of the fluorescence intensity of active β-catenin normalized with CD31 area. **(E)** Relative mRNA expression levels of Wnt/β-catenin signaling target genes: *Axin2, Lef1, Nkd1, Apcdd1, Spock2*, and *Notum* normalized with β*-actin*. Data are mean ± SD. *n* = 4–5 mice per group. Significance of data is represented as: *P < 0.05, ^**^P < 0.01, ^***^P < 0.001, ^****^P < 0.0001.

To explore how Wnt/β-catenin signaling regulates BBB permeability in adult mice, we conditionally deleted β-catenin in the brain ECs of 8–10-week-old mice. *Ctnnb1*^loxp/loxp^*; Cdh5-CreER^–^* mice were bred with *Ctnnb1*^loxp/loxp^*; Cdh5-CreER^+^* mice to generate *Ctnnb1*^loxp/loxp^*; Cdh5-CreER^–^* mice (termed as β-cat WT or WT) and *Ctnnb1*^loxp/loxp^*; Cdh5-CreER^+^* mice (termed as β-cat ECKO or ECKO) mice (Figure 1A). Oral administration of tamoxifen efficiently deleted β-catenin expression in the brain ECs of ECKO mice, but not in WT mice (Figures 1B–D).

To determine whether EC-specific deletion of β-catenin downregulates Wnt/β-catenin signaling in the brain endothelium, we evaluated mRNA expression levels of several Wnt target genes, including *Axin2, Lef1*, *Nkd1*, *Apcdd1*, *Spock2*, and *Notum*. All these Wnt target genes were downregulated in the brain tissues of β-cat ECKO mice compared with WT mice (Figure 1E). These observations suggested that β-catenin deficiency leads to significant downregulation of Wnt/β-catenin signaling in the brain endothelial cells of adult ECKO mice.

### Endothelial Cell-Specific β-Catenin Deletion Increases Blood-Brain Barrier Permeability in Adult Mice

**FIGURE 2:**
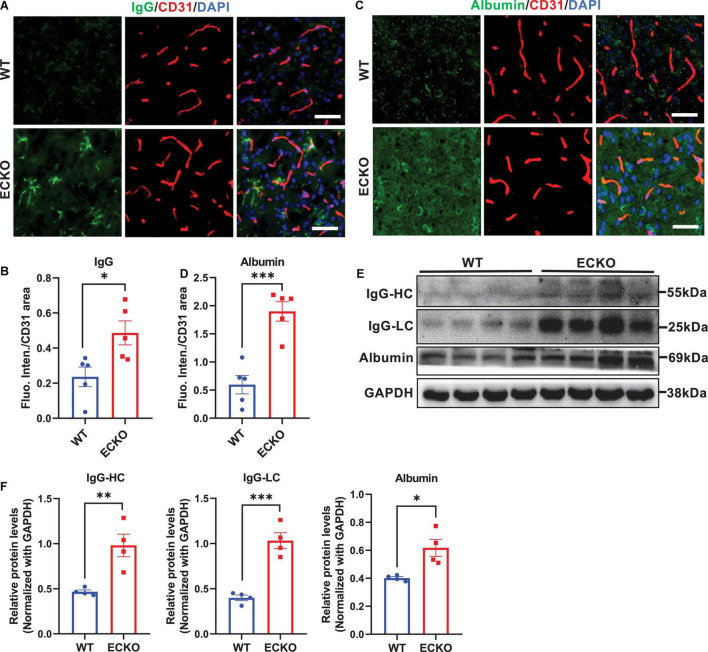
Endothelial cell-specific deletion of β-catenin increases BBB permeability in adult mice. **(A,C)** Immunofluorescence staining showing leakages of blood IgG **(A)** or albumin **(C)** in the cerebral cortex from β-cat WT mice and β-cat ECKO mice, respectively. Scale bars, 100 μm. **(B,D)** Quantifications of the fluorescence intensity of IgG **(B)** and albumin **(D)** normalized with CD31 area, respectively. **(E)** Protein levels of IgG and albumin in the cerebral cortex from β-cat WT mice and β-cat ECKO mice. **(F)** Quantification of the IgG and albumin protein levels normalized with that of GAPDH. Data are mean ± SD. *n* = 4–5 mice per group. Significance of data is represented as: *P < 0.05, ^**^P < 0.01, ^***^P < 0.001.

Genetic deficiency of endothelial β-catenin resulted in severe and prevalent leakage of plasma IgG and albumin into the cerebral cortex of ECKO mice, compared to that in WT control mice (Figures 2A–D). Western blotting of brain lysates also showed significantly increased IgG and albumin leakage into the brain tissues of ECKO mice compared to WT mice (Figures 2E,F). These data suggest that β-catenin in brain EC is indispensable to the functional integrity of the BBB in adult mice, and endothelial β-catenin deficiency causes BBB breakdown, leading to increased vascular permeability in the CNS.

### Endothelial Cell-Specific Deletion of β-Catenin Decreases Tight Junction Protein Levels and Disrupts the Intercellular Junction Ultrastructure

**FIGURE 3:**
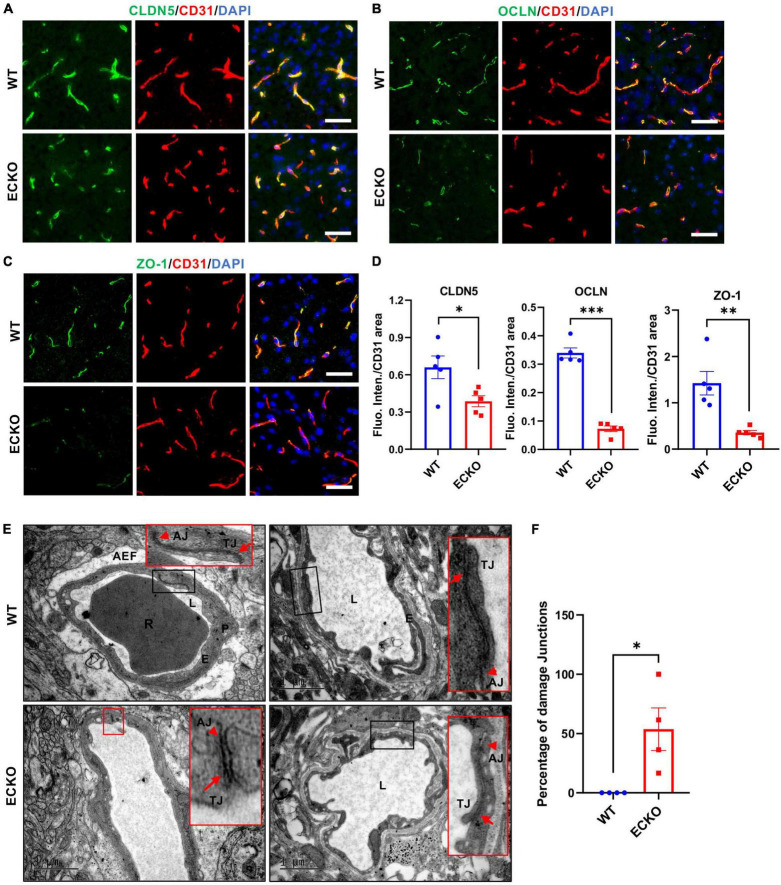
Endothelial cell-specific deletion of β-catenin decreases TJ protein levels and disrupts the intercellular junction ultrastructure. **(A–C)** Immunofluorescence staining showing the expression levels of tight junction (TJ) proteins in the cerebral cortex of β-cat WT mice and β-cat ECKO mice, respectively. Scale bars: 100 μm. **(D)** Quantifications of the fluorescence intensity of CLDN5, OCLN, and ZO-1 normalized with CD31 area, respectively. **(E)** Representative TEM images from β-cat WT control (upper panel) and β-cat ECKO mice (lower panel). Red boxed images are magnified versions of black-boxed images. Arrowheads indicate adherens junctions (AJs), and arrows indicate apical tight junctions (TJs) in both β-cat WT control and ECKO mice. Lower panel red boxed images show damaged AJs and TJs, while upper panel red boxed images show normal AJs and TJs. AEF, astrocyte end-feet; E, endothelial Cell; L, lumen; P, pericyte; R, red blood cells. Scale bars: 1 or 2 μm. **(F)** Quantification of the percentage of damaged intercellular junctions in β-cat ECKO mice compared with β-cat WT control. Data are mean ± SD. *n* = 4 mice per group, at least five microvessels per mouse were examined. Significance of data is represented as: *P < 0.05, ^**^P < 0.01, ^***^P < 0.001.

Previous studies have reported that impairment of Wnt/β-catenin signaling suppresses the expression level of TJ proteins, leading to BBB breakdown (Wang et al., 2012; Zhou et al., 2014; Tran et al., 2016). Here, we first explored the effect of endothelial β-catein deletion on TJ protein expression levels and their ultrastructure using immunofluorescence microscopy and transmission electron microscopy (TEM), respectively. β-catenin deletion in the brain endothelium resulted in significantly decreased protein levels of Claudin-5 (CLDN5), Occludin (OCLN), and ZO-1 in the cerebral cortex of ECKO mice compared with WT control mice (Figures 3A–D). In line with the reduced TJ protein levels, TEM results demonstrated obvious defects in the ultrastructure of intercellular TJ ultrastructure in ECKO mice, compared to WT mice (Figures 3E,F). Moreover, we observed disrupted adherens junctions (AJs) in ECKO mice, which were absent in WT mice (Figure 3E). This is likely due to the role of β-catenin in the direct regulation of AJs, in addition to its role in Wnt signaling (Valenta et al., 2012). The overall appearance of junctions between adjacent endothelial cells in ECKO mice had much lower electron density and larger gaps than those in WT mice (Figure 3E). Our findings suggest that compromised endothelial Wnt/β-catenin signaling downregulates TJ protein levels as well as damages TJ and AJ ultrastructure, leading to increased paracellular permeability of the BBB.

### Endothelial Cell-Specific Deletion of β-Catenin Substantially Increases Caveolae-Mediated Transcytosis *via* Downregulating Mfsd2a

**FIGURE 4:**
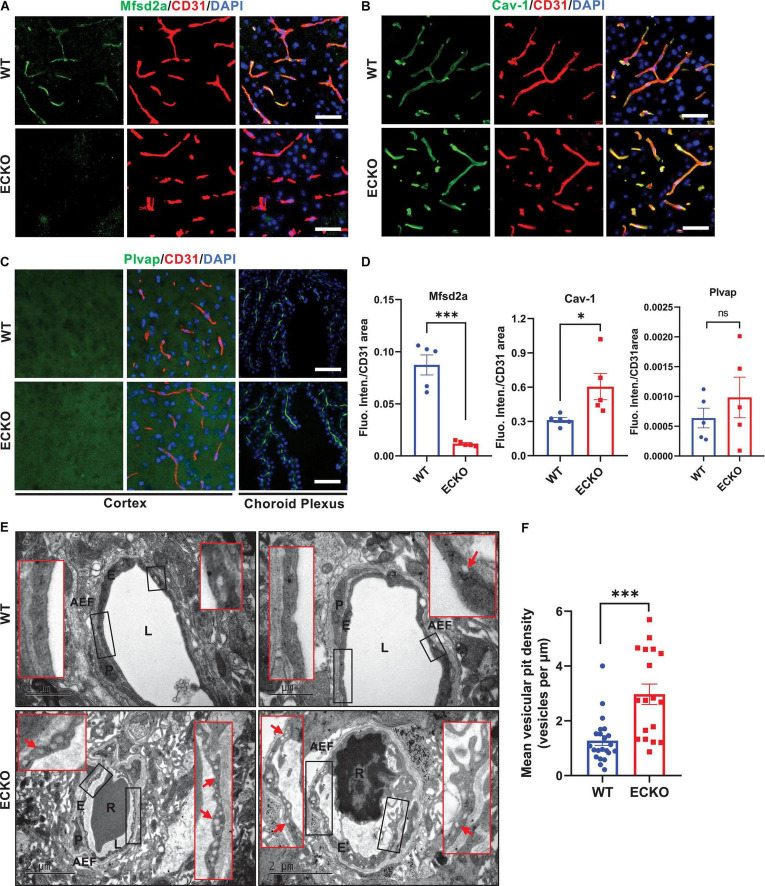
Endothelial cell-specific deletion of β-catenin substantially increases caveolae-mediated transcytosis *via* downregulating Mfsd2a. **(A,B)** Immunofluorescence staining showing the expression levels of Mfsd2a and Cav-1 in the cerebral cortex from β-cat WT mice and β-cat ECKO mice, respectively. Scale bars: 100 μm. **(C)** No Plvap staining was observed in the cerebral cortex of both β-cat WT (upper panel) and β-cat ECKO mice (lower panel). Choroid plexus was used as a positive control to show Plvap staining signal. Scale bars: 100 μm. **(D)** Quantifications of the fluorescence intensity of Mfsd2a, Cav-1, and Plvap normalized with CD31 area, respectively. **(E)** Representative TEM images from WT control (upper panel) and ECKO mice (lower panel). Red boxed images show magnified versions of black-boxed images. Arrows indicate endothelial vesicles in β-cat WT control (upper panel) and β-cat ECKO mice (lower panel). AEF, astrocyte end-feet; E, endothelial Cell; L, lumen; P, pericyte; R, red blood cells. Scale bars: 1 or 2 μm. **(F)** Quantification of mean vesicular pit density in both β-cat WT and β-cat ECKO mice. Data are mean ± SD. *n* = 4 mice per group, each data point represents one microvessel examined, totally > 18 microvessels per group. Significance of data is represented as: ns = not significant, *P* > 0.05, *P < 0.05, ^***^P < 0.001.

We next assessed whether endothelial deletion of β-catenin in ECKO mice has any impact on the transcellular permeability of the BBB and related markers. We performed immunofluorescence staining to assess whether the deletion of β-catenin in ECKO mice has any impact on the expression of Mfsd2a (a repressor of caveolae-mediated transcytosis) and Caveolin-1 (Cav-1, a marker of caveolae). In line with previous reports, Mfsd2a expression was precisely observed in the endothelium of brain microvessels and the protein colocalized with CD31 in WT mice (Figure 4A). However, in β-catenin ECKO mice, Mfsd2a expression is abrogated, suggesting an essential role of endothelial β-catenin in inducing Mfsd2a expression (Figures 4A,D). In contrast, the expression of endothelial Cav-1 was significantly upregulated in β-catenin ECKO mice, indicating augmented caveolae-mediated transcytosis (Figures 4B,D). In contrast, we did not observe any expression of Plvap, a key fenestration marker, in either WT mice or β-catenin ECKO mice (Figures 4C,D). The absence of Plvap staining signal was not due to problems related to tissue sample processing or the primary antibody, as a strong Plvap signal was observed in the choroid plexus on the same tissue sections, which is enriched with fenestrae in ECs (Figure 4C; Profaci et al., 2020).

Next, we used TEM to further assess the impact of Wnt/β-catenin signaling on endothelial transcytosis and fenestration. Our TEM analysis showed a substantial increase in transcytotic vesicles in the brain capillary ECs of β-catenin ECKO mice as compared to that of the WT controls (Figures 4E,F). The vesicles were widespread in the brain capillary ECs and included both plasma membrane-connected and free cytoplasmic vesicles, indicating a strongly augmented transcytosis rate. These vesicles were likely caveolae, as we also observed a marked increase in the caveolae marker Cav-1 (Figures 4B,D). Furthermore, we did not find any obvious fenestrae-like structure in the brain endothelium of β-catenin ECKO mice after examining 18 capillaries from five mice, consistent with the absence of Plvap protein signal (Figures 4E,F). These findings demonstrate that β-catenin plays a significant role in regulating transcellular permeability in the brain endothelium by regulating Mfsd2a expression and caveolae-mediated transcytosis.

### Wnt/β-Catenin Signaling Regulates Transcytosis-Related Genes in Brain Endothelial Cells

**FIGURE 5:**
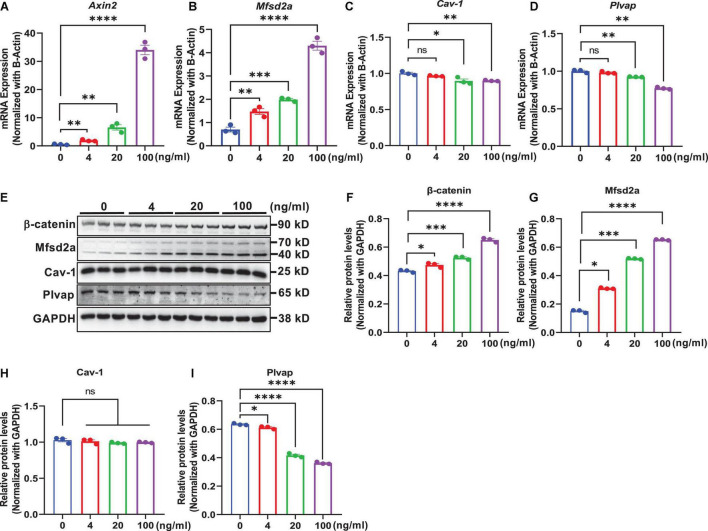
Wnt protein-induced activation of β-catenin signaling regulates transcytosis-related genes in brain endothelial cells. bEnd.3 cells were incubated with recombinant Wnt3a protein (0, 4, 20, and 100 ng/ml) for 24 h and then subjected to various measurements. **(A–D)** qRT-PCR measurement of the mRNA levels of *Axin2*, *Mfsd2a*, *Cav-1*, and *Plvap* normalized with that of β*-actin*. **(E)** Western blot measurement of β-catenin, Mfsd2a, Cav-1, and Plvap. **(F–I)** Relative protein levels of β-catenin, Mfsd2a, Cav-1, and Plvap normalized with that of GAPDH. Data are mean ± SD. *n* = 3 per group. ns = not significant, *P* > 0.05, *P ≤ 0.05, ^**^P ≤ 0.01, ^***^P ≤ 0.001, ^****^P ≤ 0.0001.

**FIGURE 6:**
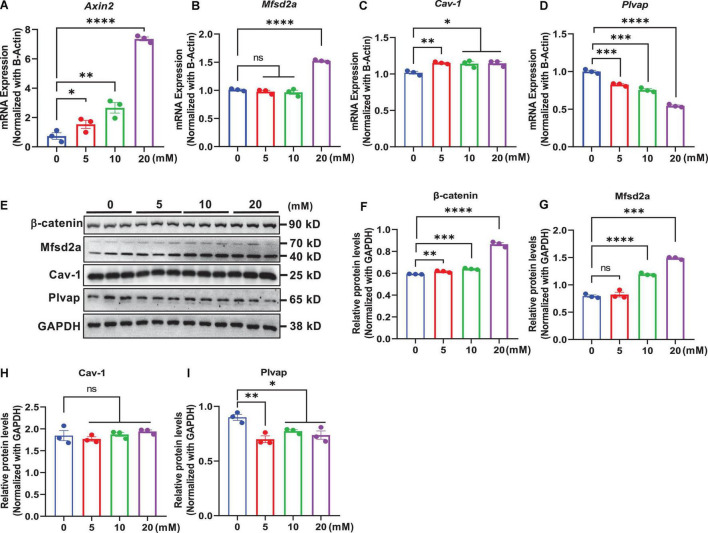
GSK-3β inhibitor-induced activation of β-catenin signaling regulates the expression of transcytosis-related genes in brain endothelial cells. bEnd.3 cells were incubated with LiCl (0, 5, 10, and 20 mM) for 24 h and then subjected to various measurements. **(A–D)** qRT-PCR measurement of the mRNA levels of *Axin2*, *Mfsd2a*, *Cav-1*, and *Plvap* normalized with that of β*-actin*. **(E)** Western blot measurement of β-catenin, Mfsd2a, Cav-1, and Plvap. **(F–I)** Relative protein levels of β-catenin, Mfsd2a, Cav-1, and Plvap normalized with that of GAPDH. Data are mean ± SD. *n* = 3 per group. ns = not significant, *P* > 0.05, *P ≤ 0.05, ^**^P ≤ 0.01, ^***^P ≤ 0.001, ^****^P ≤ 0.0001.

In an *in vitro* study, we determined whether modulation of Wnt/β-catenin signaling by exogenous treatments in mouse brain endothelial cells bEnd.3 regulated the expression of transcytosis-related genes. This was assessed by treatment with the natural ligand of canonical Wnt/β-catenin signaling activator, Wnt3a, which binds to frizzled and LRP5/6 receptors (Voloshanenko et al., 2017), and using LiCl, a small-molecule GSK-3β inhibitor that inhibits the activation of the β-catenin destruction complex (Laksitorini et al., 2019; Ji et al., 2021). We observed that the mRNA levels of *Axin2*, a commonly used Wnt target gene, were elevated in a dose-dependent manner following treatment with Wnt3a (Figure 5A) or LiCl (Figure 6A) for 24 h. Additionally, β-catenin protein levels also dose-dependently increased with Wnt3a (Figures 5E,F) or LiCl (Figures 6E,F) treatment, suggesting the stabilization of β-catenin. These results demonstrate that both Wnt3a and LiCl treatment resulted in the activation of Wnt/β-catenin signaling in brain endothelial cells.

Next, we assessed the impact of Wnt/β-catenin pathway activation on the transcytosis-related genes Mfsd2a, Cav-1, and Plvap, respectively. We found that both the mRNA and protein levels of Mfsd2a were upregulated in a dose-dependent manner upon Wnt3a or LiCl treatment (Figures 5B,E,G, 6B,E,G). These data were consistent with our observations in β-catenin ECKO mice and indicated that expression of Mfsd2a was positively correlated with Wnt/β-catenin signaling. In contrast, we observed a slight decrease in *Cav-1* mRNA levels following Wnt3a treatment, but a slight increase in *Cav-1* mRNA levels after LiCl treatment (Figures 5C, 6C). However, Cav-1 protein level remained unchanged after either Wnt3a or LiCl treatment (Figures 5E,H, 6E,H), suggesting that Cav-1 is likely not a direct target of Wnt/β-catenin signaling, and may be regulated *via* other mechanisms. Plvap expression was regulated by Wnt/β-catenin signaling at both the mRNA and protein levels as shown by either Wnt3a or LiCl treatment (Figures 5D,E,I, 6D,E,I).

## Discussion

Intercellular junctions and transcytosis are two distinct biological structures/processes and regulate the transportation of different molecules across the BBB ECs. TJs mainly control the paracellular transport, as shown in studies in Cldn5 KO and LSR KO mice (Nitta et al., 2003; Sohet et al., 2015). In contrast, endothelial transcytosis controls the transcellular transport route of both large and small molecules (Ben-Zvi et al., 2014). Here, we reported that Wnt/β-catenin signaling contributes to the regulation of BBB permeability in adults. Endothelial-specific deletion of β-catenin in adult ECKO mice compromised BBB integrity by downregulating TJ proteins (CLDN5, OCLN, and ZO-1) and damaged the ultrastructure of TJs and AJs. Furthermore, β-catenin deficiency enhanced endothelial transcytosis *via* suppressing the transcytosis inhibitor marker Mfsd2a, and upregulating caveolae-dependent transcytosis. Increased damage to the AJ and TJ ultrastructure and enhanced caveolae-mediated EC transcytosis results in BBB disruption, manifested by increased the paracellular and transcellular permeability.

Tight junction proteins (CLDN5, OCLN, and ZO-1) are overexpressed in brain ECs and regulate the paracellular permeability across the BBB (Daneman et al., 2010). Genetic deletion of β-catenin in the brain EC of adult mice downregulates claudin-1 (Cldn-1) and claudin-3 (Cldn-3) expression, without affecting the mRNA levels of other TJ proteins (Tran et al., 2016). However, we demonstrated that the protein levels of CLDN5, OCLN, and ZO-1 were downregulated in β-catenin ECKO mice, suggesting a negative impact of Wnt/β-catenin signaling inhibition on TJ proteins. Our study is consistent with the observations that endothelial β-catenin deficiency reduces CLDN5 expression in neonatal mice, and activation of Wnt/β-catenin signaling upregulated CLDN5 in the circumventricular organs (Zhou et al., 2014; Wang et al., 2019). Hence, our results suggest that Wnt/β-catenin signaling plays a key role in maintaining BBB integrity in adult mice *via* regulating CLDN5, OCLN, and ZO-1. Furthermore, AJs also contribute to cell-cell adhesion strength and paracellular permeability in addition to TJs. β-catenin not only regulates the expression of Wnt target genes *via* associating with TCF/LEF factors, but also maintains AJ complex *via* interacting with VE-cadherin and α-catenin (Valenta et al., 2012). In our β-catenin ECKO mouse model, the expressions of both AJs-associated and cytosolic/nuclear β-catenin were deleted, thus abolishing their Wnt signaling-mediating role and AJ-stabilization role. Our TEM results showed obvious damage to the ultrastructure of both AJs and TJs in β-catenin ECKO mice, revealing the significance of β-catenin in maintaining the structural integrity of BBB.

Caveolae-mediated transcytosis plays a major role in the regulation of BBB permeability under physiological and pathological conditions (Ayloo and Gu, 2019). Mfsd2a and Cav-1 have been identified as critical regulators of caveolae-mediated transcytosis (Ben-Zvi et al., 2014). High expression levels of Mfsd2a, a phospholipid transporter (Nguyen et al., 2014), repress the formation of caveolar vesicles though regulation of the membrane lipid composition of CNS endothelial cells, while Cav-1 directly participate in the formation of caveolae vesicles (Andreone et al., 2017; Parton, 2018). Our data showed that Wnt/β-catenin signaling is crucial for regulating transcytosis in the brain endothelium *via* suppressing Mfsd2a. *Mfsd2a^–/–^* mice show leaky BBB and a dramatic increase in endothelial vesicular transcytosis (Ben-Zvi et al., 2014; Andreone et al., 2017); however, these effects have not been evaluated in β-catenin-deficient mice. A recent study showed that Wnt/β-catenin signaling upregulated the expression of Mfsd2a and suppressed caveolae-mediated transcytosis in the retinal vascular endothelium (Wang et al., 2020). The impact of β-catenin on Mfsd2a expression and transcytosis has not been reported in the BBB. We found that deletion of β-catenin in the brain endothelium resulted in a nearly complete loss of Mfsd2a expression and a substantial increase in caveolae-mediated transcytosis, highlighting the essential role of β-catenin in the regulation of Mfsd2a-mediated repression of endothelial transcytosis in BBB ECs.

Transcytosis comprises both receptor/clathrin-mediated transcytosis and caveolae-mediated transcytosis (Ayloo and Gu, 2019). Clathrin-mediated transcytosis is a specific transport process that depends on the specific interactions between ligands and their receptors. However, caveolae-mediated transcytosis is a non-specific transport process that is normally repressed in the BBB. In CNS ECs, most endocytic vesicles are coated with caveolin-1 (Cav-1) (Nag, 2003; Kovtun et al., 2015), and are associated with caveolar vesicles (Williams and Lisanti, 2004). In an *in vivo* study, *Mfsd2a* genetic knockout increased in caveolae-mediated transcytosis (Andreone et al., 2017). Consistently, we also observed increased Cav-1 expression by immunostaining, and caveolar vesicles by TEM, in the brain endothelium of β-catenin-deficient mice, suggesting that the increased paracellular permeability was due to enhanced caveolae-mediated transcytosis.

The fenestration marker gene, Plvap, is not expressed in brain ECs under normal conditions. along with absence of the fenestrations in the brain ECs with BBB function. Previous studies have reported that Plvap expression is regulated by Wnt/β-catenin signaling in both brain and retinal ECs (Zhou et al., 2014), and forced activation of Wnt/β-catenin signaling in fenestrated ECs within the circumventricular organs results in decreased Plvap expression and fenestrations (Wang et al., 2019). However, we did not observe any induction of Plvap expression in the cerebral cortex and no obvious upregulation of fenestrations in the brain endothelium of β-catenin ECKO mice. This inconsistency may be related to the difference in antibodies used, mice age, or the genetic model used. We sacrificed the β-catenin ECKO mice 2 or 3 days after tamoxifen treatment, and this short time may also account for the absence of fenestrations. However, the short survival time and high mortality rate of β-catenin ECKO mice precludes a longer observation duration after the induction of endothelial β-catenin deletion (Tran et al., 2016).

**FIGURE 7:**
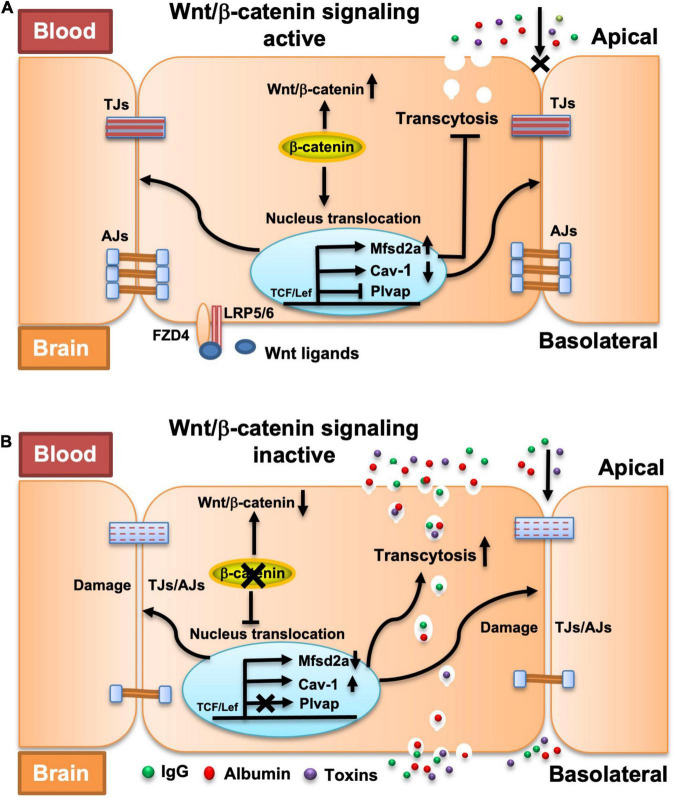
Proposed model explaining the impact of endothelial β-catenin deficiency on intercellular junctions, transcytosis, and fenestrations in BBB ECs. **(A)** In a healthy state or the presence of β-catenin, a natural Wnt ligand (Wnt3a) or GSK-3β inhibitor (LiCl) that activates the Wnt/β-catenin signaling pathway, TJs and AJs are strengthened, and endothelial transcytosis and expression of Plvap is repressed, thereby collectively maintaining the BBB integrity. **(B)** In the disease state, or with the deletion of β-catenin that compromises the Wnt/β-catenin signaling pathway, TJs and AJs are weakened and endothelial transcytosis is increased, which lead to BBB breakdown. No fenestrations and expression of Plvap are induced upon acute deletion of endothelial β-catenin.

In summary, our study provides the first evidence that β-catenin-dependent Wnt signaling maintains the integrity of the adult BBB by regulating both the intercellular junction-mediated paracellular transport route and the endothelial transcytosis-mediated transcellular transport route (Figure 7). We also revealed the mechanism by which β-catenin regulates the endothelial transcytosis in BBB ECs. The findings of this study substantially increase our understanding of the regulation of BBB integrity and also the pathogenesis of CNS diseases involving dysregulation of Wnt/β-catenin signaling, and thus, may contribute to the development of novel therapeutic strategies that manipulate the BBB and implement selective drug delivery into the CNS.

## Data Availability Statement

The original contributions presented in the study are included in the article/Supplementary Material, further inquiries can be directed to the corresponding author.

## Ethics Statement

The animal study was reviewed and approved by Shenzhen Institute of Advanced Technology, Chinese Academy of Sciences, Shenzhen, China.

## Author Contributions

JC conceived and supervised the study, analyzed the results, and revised the manuscript. BH and CF performed most of the experiments and drafted the manuscript. XH, ZF, and YY participated in tissue collection, processing, and analysis. YW commented on the manuscript. All authors approved the final version of the manuscript.

## Conflict of Interest

The authors declare that the research was conducted in the absence of any commercial or financial relationships that could be construed as a potential conflict of interest.

## Publisher’s Note

All claims expressed in this article are solely those of the authors and do not necessarily represent those of their affiliated organizations, or those of the publisher, the editors and the reviewers. Any product that may be evaluated in this article, or claim that may be made by its manufacturer, is not guaranteed or endorsed by the publisher.
