# Corrigendum: Endothelial β-catenin deficiency causes blood-brain barrier breakdown via enhancing the paracellular and transcellular permeability

**DOI:** 10.3389/fnmol.2024.1379436

**Published:** 2024-04-04

**Authors:** Basharat Hussain, Cheng Fang, Xiaowen Huang, Ziying Feng, Yuxuan Yao, Yu Wang, Junlei Chang

**Affiliations:** ^1^Shenzhen Key Laboratory of Biomimetic Materials and Cellular Immunomodulation, Institute of Biomedicine and Biotechnology, Shenzhen Institute of Advanced Technology, Chinese Academy of Sciences, Shenzhen, China; ^2^University of Chinese Academy of Sciences, Beijing, China; ^3^State Key Laboratory of Pharmaceutical Biotechnology, Department of Pharmacology and Pharmacy, Li Ka Shing Faculty of Medicine, The University of Hong Kong, Pok Fu Lam, Hong Kong SAR, China

**Keywords:** blood-brain barrier, Wnt signaling, endothelial cells, tight junctions, transcytosis

In the published article, there was an error in Supplementary Table 1. At row 5 columns 2-4 incorrect information was provided for the rabbit anti-mouse Mfsd2a antibody used for immunofluorescence (IF) staining. Row 5, “Rabbit pAb anti-mouse Mfsd2a” only included “Abcam, Cambridge, US, Cat #117618, WB, 1:500” but should have also included, “homemade rabbit polyclonal antibody using an immunogen corresponding to the mouse MFSD2A C-terminus amino acids CSDTDSTELASIL, IF, 1:500”.

The corrected Supplementary Table 1 has been published alongside the original article.

The authors apologize for this error and state that this does not change the scientific conclusions of the article in any way. The original article has been updated.

