# Supplementary material for: Endothelial β-Catenin Deficiency Causes Blood-Brain Barrier Breakdown via Enhancing the Paracellular and Transcellular Permeability
Source: Front Mol Neurosci. 2022 May 9;15:895429. doi: 10.3389/fnmol.2022.895429 (PMC9125181; doi:10.3389/fnmol.2022.895429)
Supplement: Supplementary file 1 [file Table_1.DOCX]

**Supplementary Table 1.** List of antibodies, ligands and chemical that are used in all experiments are given in table.

| Reagents | Source | Identifier | Applications | Other |
| --- | --- | --- | --- | --- |
| Antibodies |  |  |  |  |
| hamster anti-mouse CD31 | Millipore, MA, US | Cat #MAB1398Z | IF | 1:500 |
| Rabbit mAb anti-mouse active β-catenin | CST, MA, US | Cat #8814S | IF | 1:100 |
| Rabbit mAb anti-mouse total β-catenin | CST, MA, US | Cat #8480S | WB | 1:1000 |
| Rabbit pAb anti-mouse Mfsd2a | Abcam, Cambridge, US | Cat #117618 | WB | 1:500 |
|  | homemade rabbit polyclonal antibody using an immunogen corresponding to the mouse MFSD2A C-terminus amino acids CSDTDSTELASIL | | IF | 1:500 |
| Chicken pAb anti-mouse Albumin | Abcam, Cambridge, US | Cat #ab106582 | WB  IF | 1:500  1:500 |
| Rabbit anti-mouse IgG HRP Linked | CST, MA, US | Cat #7076S | WB | 1:500 |
| Rat mAb anti-mouse Plvap (MECA32) | BD Bioscience, San Diego, CA | Cat #553849 | WB | 1:500 |
| Rat mAb anti-mouse Plvap (MECA32) | Santa Cruz Biotechnology, Texas, US | Cat #SC-19603 | IF | 1:50 |
| Rabbit pAb anti-mouse Claudin-5 | Thermo Fisher, MA, US | Cat #34-1600 | WB  IF | 1:200  1:100 |
| Rabbit pAb anti-mouse Occludin | Thermo Fisher, MA, USA | Cat #40-4700 | WB  IF | 1:500  1:50 |
| Rabbit polyclonal anti-mouse ZO-1 | Proteintech, Wuhan, China | Cat #21773-1-AP | IF | 1:500 |
| Rabbit monoclonal anti-mouse ZO-1 | CST, MA, US | Cat #13663S | WB | 1:1000 |
| Rabbit mAb anti-mouse Caveolin-1 | CST, MA, US | Cat #3267S | WB  IF | 1:1000 |
| Rabbit McAb anti-mouse β-actin | Proteintech, Wuhan, China | Cat #66009-1-Ig | WB | 1:1000 |
| Rabbit McAb anti-mouse GAPDH | Proteintech, Wuhan, China | Cat #60004-1-Ig | WB | 1:1000 |
| Chemicals and recombinant proteins | | | |  |
| Wnt3a | R&D system, MN, US | Cat #5036-WN-010 | Wnt signaling | activator |
| LiCl | Sigma-Aldrich, St Louis, MO, US | Cat #213233 | GSK-3β | Inhibitor |
