# Supplementary material for: Endothelial β-Catenin Deficiency Causes Blood-Brain Barrier Breakdown via Enhancing the Paracellular and Transcellular Permeability
Source: Front Mol Neurosci. 2022 May 9;15:895429. doi: 10.3389/fnmol.2022.895429 (PMC9125181; doi:10.3389/fnmol.2022.895429)
Supplement: Supplementary file 2 [file Table_2.docx]

**Supplementary Table 2.** Primer sequences that are used to determine mRNA levels for all genes are listed.

| 1 | Ms-Cav-1 | Forward | 5’ CCGTGCATCAAGAGCTTCCT 3’ |
| --- | --- | --- | --- |
|  |  | Reverse | 5’ CTCTTTCTGCGTGCTGATGC 3’ |
| 2 | Ms-Plvap | Forward | 5’ GCTGGTACTACCTGCGCTATT 3’ |
|  |  | Reverse | 5’ CCTGTGAGGCAGATAGTCCA 3’ |
| 3 | Ms-Mfsd2a | Forward | 5’ CTCCTGGCCATCATGCTCTC 3’ |
|  |  | Reverse | 5’ GGCCACCAAGATGAGAAA 3’ |
| 4 | Ms-Axin2 | Forward | 5’ GCCGACCTCAAGTGCAAACTC 3’ |
|  |  | Reverse | 5’ GGCTGGTGCAAAGACATAGCC 3’ |
| 5 | Ms-Lef1 | Forward | 5’ CCACCTTCTACCCCCTGTCT 3’ |
|  |  | Reverse | 5’ GACATGGAAGTGTCGCCTGA 3’ |
| 6 | Ms-Abcd1 | Forward | 5’ CGCCTGGAGGGCTTTCAAG 3’ |
|  |  | Reverse | 5’ GGACCCGACCTTACTTCACAG 3’ |
| 7 | Ms-Nkd1 | Forward | 5’ AGGAAAGGCATCGAGGAGTG 3’ |
|  |  | Reverse | 5’ TCGCTCAGTCTCTCCATTCTC 3’ |
| 8 | Ms-Spock2 | Forward | 5’ CATCCGCCCCTTCTTCAACT 3’ |
|  |  | Reverse | 5’ ACAGCTCGGGATGAAGACAC 3’ |
| 9 | Ms-Notum | Forward | 5’ CGAGAACTGCGTGGTACACT 3’ |
|  |  | Reverse | 5’ TGGCCTTATGGCTGTCATGG 3’ |
| 10 | Ms-Claudin-5 | Forward | 5’ GCAAGGTGTATGAATCTGTGCT 3’ |
|  |  | Reverse | 5’ GTCAAGGTAACAAAGAGTGCCA 3’ |
| 11 | Ms-Occludin | Forward | 5’ TTGAAAGTCCACCTCCTTACAGA 3’ |
|  |  | Reverse | 5’ CCGGATAAAAAGAGTACGCTGG 3’ |
| 12 | Ms-ZO1 | Forward | 5’ GCCGCTAAGAGCACAGCAA 3’ |
|  |  | Reverse | 5’ TCCCCACTCTGAAAATGAGGA 3’ |
| 13 | β-Actin | Forward | 5’ GAAGTGTGACGTTGACATCCG 3’ |
|  |  | Reverse | 5’ GTCAGCAATGCCTGGGTACAT 3’ |
| F: Forward direction; R: Reverse direction | | | |
